# FOXR2-activated CNS neuroblastoma: Characterized by variable structural disruption of the *FOXR2* regulatory region, recurrent copy number alterations, and elevated *FOXR2* expression

**DOI:** 10.1093/neuped/wuag014

**Published:** 2026-03-19

**Authors:** Jianling Ji, Venkata Yellapantula, Dong Xu, Dolores Estrine, Alexander Markowitz, Jennifer Han, Katherine Ma, Cindy Fong, Debra Hawes, Ashley Margol, Katrina O’Halloran, Nathan J Robison, Jaclyn A Biegel, Jennifer A Cotter, Gordana Raca, Jessica W Tsai

**Affiliations:** Department of Pathology and Laboratory Medicine, Children’s Hospital Los Angeles, Los Angeles, CA, United States; Department of Pathology, Keck School of Medicine of University of Southern CA, Los Angeles, CA, United States; Department of Pathology and Laboratory Medicine, Children’s Hospital Los Angeles, Los Angeles, CA, United States; Department of Pathology, Keck School of Medicine of University of Southern CA, Los Angeles, CA, United States; Department of Pathology and Laboratory Medicine, Children’s Hospital Los Angeles, Los Angeles, CA, United States; Department of Pathology and Laboratory Medicine, Children’s Hospital Los Angeles, Los Angeles, CA, United States; Department of Pathology and Laboratory Medicine, Children’s Hospital Los Angeles, Los Angeles, CA, United States; Department of Pathology and Laboratory Medicine, Children’s Hospital Los Angeles, Los Angeles, CA, United States; Department of Pathology, Keck School of Medicine of University of Southern CA, Los Angeles, CA, United States; Department of Pediatrics, Keck School of Medicine of University of Southern CA, Los Angeles, CA, United States; Cancer and Blood Disease Institute, Children’s Hospital Los Angeles, Los Angeles, CA, United States; Department of Pathology and Laboratory Medicine, Children’s Hospital Los Angeles, Los Angeles, CA, United States; Department of Pathology, Keck School of Medicine of University of Southern CA, Los Angeles, CA, United States; Department of Pathology and Laboratory Medicine, Children’s Hospital Los Angeles, Los Angeles, CA, United States; Department of Pathology, Keck School of Medicine of University of Southern CA, Los Angeles, CA, United States; Department of Pathology and Laboratory Medicine, Children’s Hospital Los Angeles, Los Angeles, CA, United States; Department of Pathology, Keck School of Medicine of University of Southern CA, Los Angeles, CA, United States; Department of Pediatrics, Keck School of Medicine of University of Southern CA, Los Angeles, CA, United States; Cancer and Blood Disease Institute, Children’s Hospital Los Angeles, Los Angeles, CA, United States; Saban Research Institute, Children’s Hospital Los Angeles, Los Angeles, CA, United States; USC Norris Comprehensive Cancer Center, Epigenetic Regulation in Cancer Program, Los Angeles, CA, United States

**Keywords:** CNS neuroblastoma, FOXR2, methylation profiling, optical genome mapping, copy number abnormality

## Abstract

**Background:**

FOXR2 is a forkhead box transcription factor implicated in central nervous system neuroblastoma (CNS-NB FOXR2). The underlying genetic mechanisms and clinical features of CNS-NB FOXR2 have yet to be elucidated.

**Methods:**

Six CNS-NB FOXR2 cases were identified through DNA methylation profiling. Optical genome mapping, chromosomal microarray, whole genome sequencing, OncoKids panel, RNA sequencing (RNA-seq), and clinical data were analyzed.

**Results:**

Case 1 demonstrated a 13-kb insertion approximately 25 kb upstream of *FOXR2*. Case 2 showed 2 non-contiguous focal gains in Xp11.21 (encompassing *FOXR2*) and Xp22.2p22.13, approximately 37 Mb apart, resulting from a complex rearrangement disrupting the *FOXR2* regulatory region. Case 3 harbored a pericentric inversion between *RLIM* and a site 17.98 kb upstream of *FOXR2*. Recurrent copy number alterations included 1q gain (100%), 16q loss (80%), distal 11q loss (60%), and gain of chromosomes 8 and 17q (40% each). All three female patients showed X chromosome loss. *FOXR2* expression was elevated in both cases with available RNA-seq data.

Overall survival ranged from 1.81-15.62 years. Clinical treatment was highly variable, and molecular testing was unavailable at diagnosis for 4 of 5 patients.

**Conclusions:**

Structural disruption of the *FOXR2* regulatory region, recurrent copy number alterations, and elevated *FOXR2* expression are features of CNS-NB FOXR2. These rearrangements do not alter the coding region and may not be detected by routine testing. Initial pathology may suggest other entities, highlighting the importance of molecular testing at diagnosis. Favorable clinical outcomes require accurate initial diagnosis as evidenced by our clinical cohort.

Key points

- CNS NB-FOXR2 tumors show diverse structural alterations upstream of *FOXR2*, including insertion, inversion, complex rearrangement, duplication, and deletion.
- A characteristic copy number profile (1q gain, 16q loss, 11q loss, chr8 gain, 17q gain, X loss in females) may aid differential diagnosis.

Importance of the study

Central nervous system (CNS) NB-FOXR2 is a newly defined pediatric brain tumor entity that is challenging to diagnose due to histologic overlap with other high-grade CNS tumors. This study identifies novel, non-recurrent structural alterations upstream of *FOXR2* that drive its activation, many of which are undetectable by standard clinical testing. We also describe a recurrent pattern of copy number changes that may serve as diagnostic clues. Compared to prior literature, our work expands the molecular landscape of CNS NB-FOXR2 and emphasizes the need for diagnostic characterization using advanced genomic tools such as optical genome mapping and whole genome sequencing with structural variant analysis. These findings have important translational implications for improving diagnostic accuracy, guiding treatment decisions, and refining molecular workflows for rare pediatric brain tumors.

## Introduction

Forkhead box R2 (FOXR2) is a forkhead transcription factor^1^^,^^2^ located on the X chromosome that has been recently implicated as a potent oncogenic driver in multiple pediatric cancers, including diffuse midline gliomas,^3^ medulloblastomas,^4^^,^^5^ peripheral nerve sheath tumors,^6^ and the rare, relatively new tumor entity central nervous system neuroblastoma with FOXR2 activation (CNS NB-FOXR2).^7-9^

Embryonal tumors of the CNS have undergone multiple classification revisions and were previously referred to by the now-outdated term, “primitive neuroectodermal tumors” (CNS-PNET). However, given significant heterogeneity among these tumors,^10^ in 2016, extensive molecular characterization was performed analyzing DNA methylation profiles of over 300 CNS-PNET, resulting in the identification of CNS NB-FOXR2 as a new molecular entity,^7^ officially recognized by the World Health Organization (WHO) in 2021.^11^ Histopathologically, these tumors are usually composed of round cells with embryonal to neurocytic morphology and are immunoreactive for OLIG2, synaptophysin, and TTF-1, although studies suggest this immunoexpression is highly variable from case to case.^12^ Prior molecular analysis based on RNA sequencing (RNA-seq) highlighted SOX10 and Ankyrin Repeat Domain 55 (ANKRD55) expression as potential histologic markers for CNS NB-FOXR2,^12^ while others have shown diagnostic utility with an immunohistochemistry panel comprised of OLIG2, synaptophysin, and SOX10 coupled with FISH for the detection of 1q gain.^13^ Morphological diagnosis alone is insufficient for CNS NB-FOXR2.

Accurate diagnosis of CNS NB-FOXR2 requires molecular analysis, most commonly through DNA methylation profiling, which is commonly performed on pediatric brain tumors. In some instances, RNA-seq can provide additional transcriptomic information, particularly since *FOXR2* expression is normally undetectable in all tissue except the testis.^3^ However, elevated *FOXR2* expression is not exclusive to CNS NB-FOXR2 and has also been observed in other CNS tumor types, including subsets of diffuse midline gliomas^3^ and medulloblastomas.^14^ Recent work demonstrates that CNS NB-FOXR2 tumors originate from LHX6+/DLX+ interneuron lineages.^8^^,^^9^ Previous studies have identified structural abnormalities in CNS NB-FOXR2, including JMJD1C-*FOXR2* interchromosomal translocation,^7^ tandem duplications involving *FOXR2*, and a mitochondrial-nuclear insertion leading to a novel promoter^14^ (**Table 1**); however, these findings appear to be non-recurrent and likely represent a limited set of structural alterations, with additional variants yet to be discovered. Importantly, these rearrangements typically affect regulatory regions upstream of *FOXR2*, including recently identified novel promoters,^3^^,^  ^15^ rather than the *FOXR2* coding sequence. Complex structural rearrangements involving non-coding regions of the genome can often be challenging to detect by routine clinical diagnostic methods, which typically focus on protein-coding regions of the genome, or utilize custom panels that may not include *FOXR2*. Moreover, the clinical outcome data for these patients have been rather limited,^12^^,^^14^^,^^16^^,^^17^ and further information is needed regarding their treatment regimens, extent of surgical resection, use of irradiation, therapy at relapse, and survival.

**Table 1:** Structural alterations affecting *FOXR2* in CNS neuroblastoma: published and study cases.

Reference	Tumor	Method of detection	Alterations		Number
Sturm et al Cell 2016	CNS-NB with FOXR2 activation	Whole genome sequencing, RNA sequencing	*JMJD1C*-*FOXR2*	Interchromosomal rearrangement	1
			*LOC550643*-*FOXR2*	Tandem duplication	1
			*JPX*-*FOXR2*	Tandem duplication	1
			Intergenic deletion between *FOXR2* and *MAGEH1*	Deletion	2
			*MT-CYB*	Mitochondrial-nuclear insertion, novel promoter	1
Siskar et al Neuro Oncology 2025 (6 cases in comut plot)	CNS-NB with FOXR2 activation	Whole genome sequencing, RNA sequencing	*RBM10*-*FOXR2* (Exon -6)	Fusion, novel promoter	1
			*BCOR*-*FOXR2* (Exon -6)	Fusion, novel promoter	1
			*MARCHF6*-*FOXR2* (Exon -6)	Fusion, novel promoter	1
			*USP51*-*FOXR2* (Exon -6)	Fusion, novel promoter	1
			Exon -7	Novel promoter only	1
			LINE-1 insertion	LINE-1 insertion, novel promoter	1
Cases included in this study	CNS-NB with FOXR2 activation (Cases 1, 2, and 6)	Optical genome mapping	Insertion with breakpoint upstream *FOXR2*	Insertion	1
			Complex rearrangement with focal gains in Xp11.21 and Xp22.2p22.13 with breakpoint at upstream *FOXR2*	Complex rearrangement	1
		Whole genome sequencing	Inversion involving *RLIM* and *FOXR2*, with breakpoint upstream of *FOXR2*	Pericentric inversion	1

We aimed to further investigate structural alterations in CNS NB-FOXR2 using optical genome mapping (OGM), an approach that enables more reliable detection of structural variants (SVs). Additionally, we summarized other molecular findings from our cohort, including copy number variants and RNA expression profiles, along with clinical characteristics. To complement our in-house cases, we also incorporated available whole genome and RNA-seq data from a CNS NB-FOXR2 case in the Children’s Brain Tumor Network (CBTN).

## Materials and methods

### Cases included in this study

The in-house pathology database and the CBTN database were searched for cases of CNS NB-FOXR2. Inclusion criteria included methylation profiling results and/or a final integrated molecular pathology diagnosis matching CNS NB-FOXR2. A total of 6 cases meeting these criteria were identified, 5 from the in-house database and one from CBTN. All 6 cases had an integrated diagnosis of CNS NB-FOXR2, with 5 showing methylation profiling results consistent with this diagnosis. This study was reviewed and approved by the Institutional Review Board of the Children’s Hospital Los Angeles and the University of Southern California (CHLA-25-00173); patients’ informed consent/assent/permission for the use of coded samples and extracted clinical information was waived. This study complies with all relevant ethical regulations, including the Declaration of Helsinki.

### Clinical annotation

Clinical features were extracted from medical records, including clinical course, demographic information, follow-up duration, pathology reports, survival outcomes, and treatment regimens (chemotherapy, radiation, and surgery). Disease recurrence or progression was defined as either clinical or radiographic progression. The time to each recurrence was defined as the time from initial diagnosis to recurrence. Metastatic disease was assessed using MRI of the spine.

### Optical genome mapping

Of the 5 in-house CNS-NB FOXR2 cases, 2 (Cases 1 and 2) had frozen samples available for OGM. Approximately 15 mg of frozen tissue per sample was homogenized using Tissueruptor II (Qiagen, Germantown, MD), digested with proteinase K, and lysed using Bionano Genomics (San Diego, CA) reagents. High molecular weight genomic DNA was precipitated onto Nanobind magnetic disks with isopropanol, washed with ethanol-based buffers, and eluted in buffer. DNA concentration was measured using the Promega Quantus Fluorometer with the QuantiFluor ONE dsDNA System. OGM was performed according to the manufacturer’s protocols. Briefly, fluorescent labeling was achieved by enzymatic incorporation of fluorophores at specific 6-base pair recognition motifs. Labeled DNA was counterstained, linearized in nanochannels, and imaged on the Bionano Saphyr system. The resulting high-resolution genome maps were aligned to the GRCh38/T2T reference genome to identify SVs. Both Rare Variant and De Novo assembly pipelines were used to detect somatic and complex SVs, with variant calls supported by consensus from multiple molecules. Data analysis was performed using Bionano Access software (Bionano Genomics).

### WGS analysis for the CBTN case

Whole genome sequencing data on the tumor sample was downloaded from CBTN (Case 6). SVs were identified using the GATK-SV pipeline, which integrates multiple tools, including Manta, WHAM, GATK-CNV, CNVkit, and cn. MOPS.^18^ All SV calls were subsequently reviewed for relevance and accuracy. Relevant variant calls were subsequently confirmed through Integrative Genomics Viewer (IGV) inspection using the BAM files provided by CBTN.

### Chromosomal microarray analysis

Chromosomal microarray analysis (CMA) was performed on 4 cases (Cases 1, 2, 4, and 5) using either the OncoScan or CytoScan HD platforms (Thermo Fisher Scientific, Waltham, MA), according to the manufacturer’s protocols. Copy number variants were analyzed using Chromosome Analysis Suite (Thermo Fisher Scientific) and NxClinical 6.0 (Bionano Genomics). Detected copy number abnormalities were reviewed and reported if they were found to have clear or suspected clinical relevance.

### DNA methylation array analysis

Genomic DNA was extracted from formalin-fixed paraffin-embedded (FFPE) using the QIAamp DNA FFPE Tissue Kit, followed by bisulfite conversion using EZ DNA Methylation-Lightning kit (Zymo Research, Irvine, CA). FFPE-derived DNA was repaired using the Infinium HD FFPE restore protocol and processed with the Infinium MethylationEPIC kit per manufacturer’s protocol (Illumina, San Diego, CA). Of the 5 in-house cases, 4 had methylation profiling performed (Cases 1, 2, 3, and 5); the CBTN case was processed through our CHLA CPM MAAv2.0 pipeline, which utilizes an ensemble of machine-learning models, including random forest and k-nearest neighbor, to predict CNS tumor superfamily, family, and class. UMAP analysis was also performed to visualize clustering patterns and assess tumor classification separation for CNS embryonal tumors.^19^

### RNA-sequencing

An exome capture-based transcriptome sequencing (RNA-seq) assay was performed on 2 in-house CNS-NB-FOXR2 cases (Cases 1 and 5), following the manufacture’s protocols^20^ (Twist Bioscience, San Francisco, CA). *FOXR2* expression (log2-transformed TPM values) was evaluated in 2 in-house cases and 1 case from the CBTN (Case 6) and compared with expression data across multiple cancer types from the Treehouse Childhood Cancer Initiative. A summary of molecular testing performed for each case is provided in **Table 2**.

**Table 2:** Patient characteristics and summary of molecular testing performed.

						n = 6	
Age at diagnosis							
Median (years)						3.49	
Range (years)						1.08-9.42	
Sex							
Female						4	
Male						2	
Tumor location							
Frontal lobe						1	
Parietal lobe						1	
Temporal lobe						3	
Frontal and temporal lobes						1	
Metastasis							
Metastatic						0	
Non-metastatic						6	
Surgery extent at diagnosis							
Gross total resection						2	
Subtotal resection						2	
Biopsy only						1	
No surgery						1	
Lines of chemotherapy							
0						0	
1						2	
2						1	
3						2	
4						1	
Radiation at diagnosis							
No						3	
Yes						3	
Number of relapses/progression							
0						1	
1						1	
2						2	
3						0	
4						1	
Median follow up from initial diagnosis						9.16 years	
Range of overall survival from initial diagnosis						1.81-17.41 years	
Range of time to first progression from initial diagnosis						0.36-2.84 years	
Case ID	Sex	Methylation	OncoKids	CMA	RNA-seq	OGM	WGS
1	F	X	X	X	X	X	
2	F	X		X		X	
3	F	X					
4	F			X			
5	M	X	X	X	X		
6	M	X			X		X

X indicates test performed on the tumor sample; blank indicates test not performed.

## Results

### Clinical characteristics

Within our cohort of CNS NB-FOXR2 patients at CHLA, we had 5 total patients: 4 female (Patient 1-4) and 1 male (Patient 5). One additional male patient (Patient 6) was added to the cohort from CBTN for a total of 6 individuals. Overall survival ranged from 1.81-15.62 years, with 1 patient deceased 2.08 years following initial diagnosis (**Table 2**). Age at initial diagnosis ranged from 1.08-9.42 years. Primary tumor locations included left temporal lobe (n = 3), right parietal lobe (n = 1), right frontal lobe (n = 1), and both frontal and temporal lobes (n = 1). None of the patients had metastatic diseases at diagnosis. Three of 5 patients received radiation at diagnosis, although this was highly variable, including 23.4 Gy CSI with 32 Gy focal boost, 54 Gy focal radiation, and 55.8 Gy focal proton radiation. Four of 5 received chemotherapy at initial diagnosis. All patients in the cohort received at least one line of chemotherapy throughout their clinical course, however, the types of chemotherapy were highly variable. At diagnosis, 2 patients received sub-total resection (STR), while 2 had gross total resection (GTR). One patient had only a biopsy at the time of diagnosis, while 1 patient did not have surgery initially due to the impression of a low-grade tumor by imaging then subsequently had an STR at the time of first progression. Time to first recurrence/progression ranged from 0.35-2.84 years (n = 4). Notably, 4 of 5 CHLA patients did not have FOXR2-activated CNS neuroblastoma as their initial diagnosis due to lack of molecular testing (unknown for the CBTN case). Importantly, molecular testing was performed later in their treatment courses, likely accounting for the highly variable treatment approaches and highly variable clinical outcomes. In one case (Patient 5), initial treatment for presumed high grade glioma was modified to include craniospinal irradiation and maintenance chemotherapy following molecular confirmation of CNS NB-FOXR2, highlighting the critical importance of early molecular testing.

### Pathology summary

The initial diagnoses from the pathology reports were highly variable, including the following: primitive neuroectodermal tumor (PNET) in Case 1; glioblastoma, small cell variant in Case 2; CNS embryonal tumor NOS (Case 3); anaplastic oligoastrocytoma in Case 4; and CNS neuroblastoma FOXR2-activated in Case 5. The case from CBTN (case 6) was initially diagnosed as a CNS embryonal tumor without a specific subtype. Case 5 underwent DNA methylation profiling, OncoKids panel testing,^21^ and CMA analysis, which established a definitive diagnosis of CNS NB-FOXR2 at presentation.

### Molecular findings

#### Structural alterations detected by OGM and whole genome sequencing.

**Figure 1:**
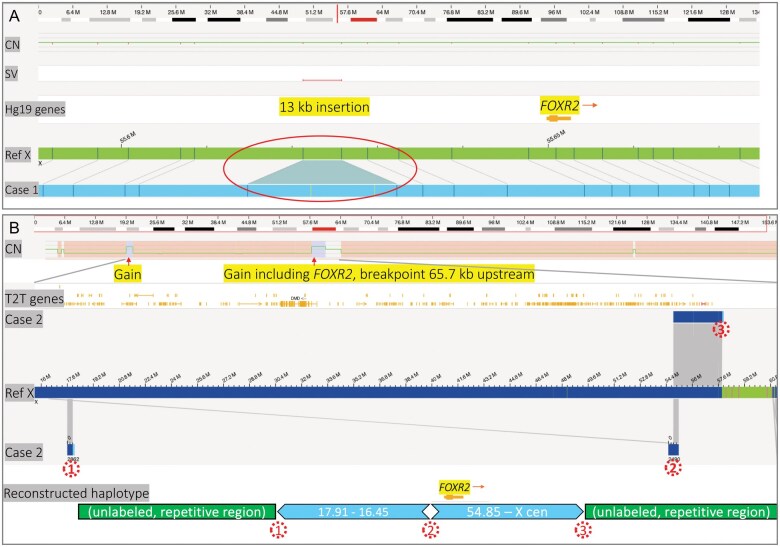
Optical genome mapping of case 1 and case 2. (A) Case 1 shows a 13-kb insertion approximately 25 kb upstream of the *FOXR2* gene on the X chromosome. This insertion is located within the regulatory region of *FOXR2* but does not disrupt its coding sequence. (B) OGM of Case 2 reveals a complex structural rearrangement involving upstream of *FOXR2*. Two non-contiguous focal copy number gains were identified on Xp11.21 and Xp22.2p22.13 (∼37 Mb apart). The proximal gain includes *FOXR2*, with a duplication breakpoint located 65.7 kb upstream. The distal duplication is inverted and fused with the proximal gain at the breakpoint, 65.7 kb upstream of *FOXR2* (labeled as breakpoint 2 in the figure). The resulting haplotype contains fused, inverted duplications flanked by unlabeled repetitive regions of unknown origin, consistent with a complex inter- or intra-chromosomal rearrangement affecting *FOXR2* regulatory elements. Rearrangement breakpoints are indicated by red dotted circles and labeled numerically (1, 2, 3) to correspond to structural variant positions. CN: copy number; SV: structural variant.

—Case 1 showed a 13-kb insertion located approximately 25 kb upstream of the *FOXR2* gene on the X chromosome by OGM analysis (**Figure 1A**, **Table 1**). This insertion maps in the putative regulatory region of *FOXR2* but does not disrupt the coding sequence, suggesting a potential impact on gene expression through alteration of upstream regulatory elements.

Case 2 demonstrated loss of 1 X chromosome, along with 2 noncontiguous focal copy number gains on Xp11.21 and Xp22.2p22.13, approximately 37 Mb apart, detected by chromosomal microarray. The proximal gain included the *FOXR2* locus, with a duplication breakpoint located 65.7 kb upstream of the gene. Subsequent OGM revealed that the distal duplication was inverted and fused with the proximal gain at the second breakpoint. The *FOXR2* gene is near this second breakpoint, positioned 65.7 kb upstream. The resulting haplotype contains the 2 copy number gains fused in opposite orientation and flanked by unlabeled repetitive regions of unknown origin. The structural configuration is consistent with a complex inter- or intra-chromosomal rearrangement that likely disrupts the *FOXR2* regulatory region and may contribute to aberrant gene activation (**Figure 1B**, **Table 1**).

Case 6 was analyzed using whole genome sequencing of the tumor, which revealed a pericentric inversion involving the *RLIM* gene at Xq13.2 and the *FOXR2* gene at Xp11.21. The inversion breakpoints were mapped to a position approximately 17.98 kb upstream of *FOXR2* and within intron 1 of ring finger protein, LIM domain interacting (*RLIM*) (RefSeq: NM_016120.4). These structural alterations were confirmed by visual inspection using IGV (**Figure S1**, **Table 1**). The rearrangement brings a portion of the *RLIM* gene into proximity with regulatory elements of *FOXR2*, potentially altering the transcriptional regulation of *FOXR2*.

We also summarized the structural alterations affecting the *FOXR2* upstream regulatory region in CNS NB-FOXR2 in **Table 1**, including three cases from our own cohort.

#### Copy number alterations in CNS NB-FOXR2.

**Figure 2:**
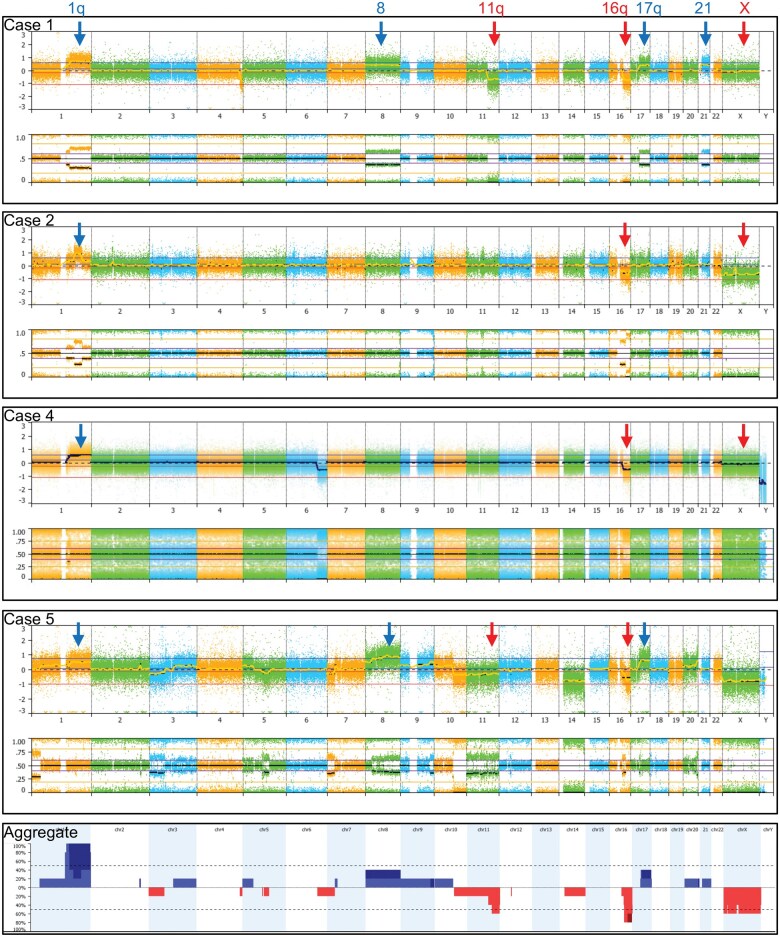
Copy number alterations identified in CNS NB-FOXR2. Whole genome view of cases with available chromosomal microarray (CMA) data. Cases 1, 2, 4, and 5 are shown from top to bottom. Copy number gains observed in more than 2 cases are indicated by blue arrows. Recurrent copy number losses are indicated by red arrows. The bottom panel shows aggregated copy number alterations, combining data from the four CMA cases above and one additional case with copy number data derived from methylation array analysis. Dark blue represents high-level copy number gains (>3 copies), and dark red indicates homozygous deletions.

—Copy number variant analysis was performed using data from 4 in-house CMA cases and 1 case with methylation CNV plot available. All 5 cases (100%) showed a copy number gain involving chromosome 1q. In 4 cases, the gain encompassed the entire long arm of chromosome 1 (1q), with 1 of these also extending into the short arm (1p). The remaining 1 case showed a near-complete 1q gain, with the pericentric 1q region retaining two copies. Loss of 16q material was observed in 4 out of 5 cases (80%). One case demonstrated a complete loss of the long arm, while the other 3 showed loss of most of 16q. All 16q losses were terminal rather than interstitial. Losses involving 11q were identified in 3 of the 5 cases (60%), all affecting the distal half of 11q. Interestingly, loss of the X chromosome was detected in all 3 female patients, whereas the male patient analyzed by CMA exhibited a normal X chromosome copy number. CMA analysis also revealed that 3 out of 4 cases showed no copy number alterations involving the *FOXR2* gene. Other recurrent alterations included gains of chromosome 8 and most of 17q (**Figure 2**). Non-recurrent copy number alterations included losses or loss of heterozygosity in 1p, 3p, 4q, 5q, 6q, 7p, 10q, and 14, and gains in 2q, 3q, 5p, 7p, 9, 10p, 20q, and 21q. Among the 5 cases with copy number data available in this series, most had alterations involving a limited number of chromosomes, whereas Case 5 demonstrated more complex CNVs, affecting more than 10 chromosomes and/or chromosomal segments (**Figure 2**).

#### Methylation clustering, FOXR2 expression, and other molecular findings.

**Figure 3:**
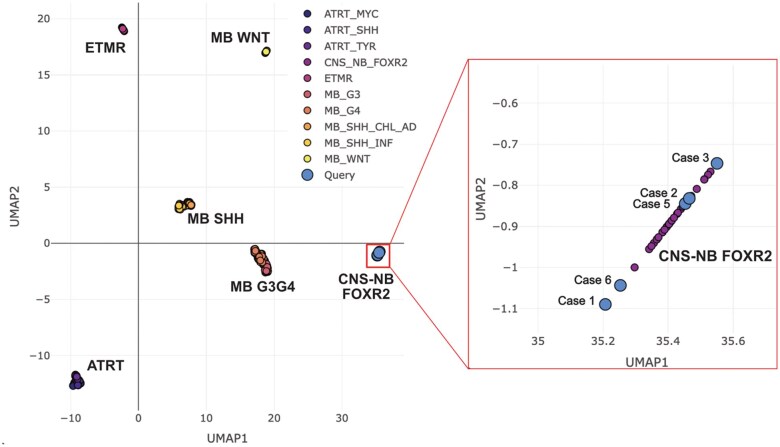
UMAP clustering of DNA methylation profiles derived from CNS embryonal tumors. (A) CNS-NB FOXR2 forms a distinct cluster (red box) and is separable from other CNS embryonal tumors, including medulloblastoma (MB), ETMR, and AT/RT. (B) Cases 1, 2, 3, 5, and 6 have available methylation data and cluster with the CNS NB-FOXR2 reference cohort.

—DNA methylation profiling showed that all five cases were classified as CNS-NB FOXR2, with high calibrated scores. UMAP analysis showed that the tumors formed a distinct cluster overlapping with the reference CNS-NB FOXR2 dataset, and were clearly separable from other CNS embryonal tumors, including medulloblastoma, embryonal tumor with multilayered rosettes (ETMR), and atypical teratoid/rhabdoid tumor (AT/RT) (**Figure 3**).

**Figure 4:**
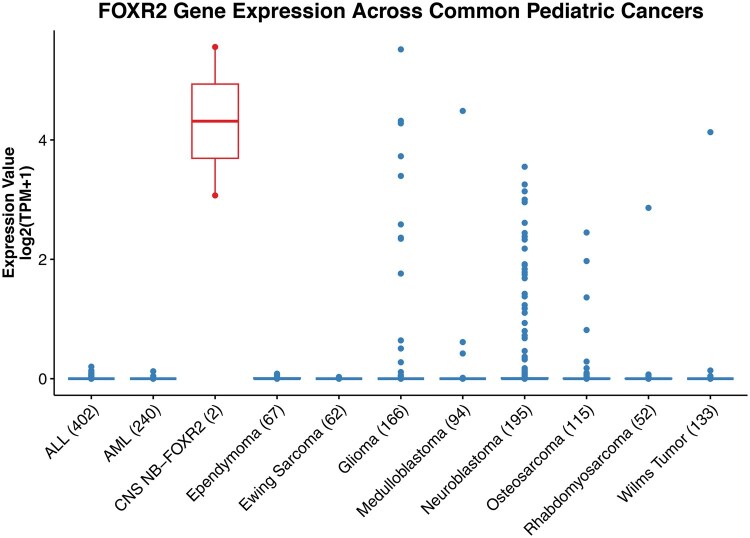
*FOXR2* gene expression across pediatric cancers. CNS NB-FOXR2 cases (n = 2, shown in red) exhibit elevated FOXR2 expression compared to other tumor types, including ALL, AML, ependymoma, and Ewing sarcoma. Notably, elevated FOXR2 expression is also observed in subsets of glioma, neuroblastoma, and other pediatric cancers.

RNA-seq analysis did not reveal any *FOXR2* fusions but demonstrated elevated *FOXR2* expression in CNS NB-FOXR2 cases. Of the 3 cases with available RNA-seq data (Cases 1 and 5, and 1 from CBTN), Case 5 did not meet the QC thresholds (total supporting reads <50 million), and was excluded from expression analysis. The remaining 2 cases (n = 2, shown in red in **Figure 4**) exhibited high *FOXR2* transcript levels, with log_2_-transformed TPM values of 5.56 and 3.07, respectively. These values are higher than those observed in some pediatric tumor types, including acute lymphoblastic leukemia (ALL), acute myeloid leukemia (AML), ependymoma, and Ewing sarcoma, which generally show no or negligible *FOXR2* expression. Notably, elevated *FOXR2* expression was also observed in subsets of other pediatric cancers, such as gliomas and neuroblastomas (**Figure 4**), consistent with the previous findings that *FOXR2* activation may occur across a broader spectrum of tumors. The expression levels in CNS NB-FOXR2 cases appeared to be at the higher end of the spectrum, supporting the hypothesis that structural rearrangements involving *FOXR2* contribute to its transcriptional upregulation and oncogenic activity in this tumor type.

Notably, no clinically significant DNA sequence variants or gene fusions were identified in the 2 in-house cases (Cases 1 and 5) by the OncoKids panel, which does not include *FOXR2* in its DNA variant or RNA fusion design. In the CBTN case, whole genome sequencing analysis did not reveal any clinically significant DNA sequence variants, although a pericentric inversion involving regulatory region of *FOXR2* was detected as previously described.

## Discussion

CNS NB-FOXR2 is a rare pediatric brain tumor entity that presents significant challenges to accurate workup and diagnosis.^13^^,^  ^22^ In our cohort, initial pathological diagnoses varied considerably; without molecular testing including DNA methylation upfront, cases were either misclassified or lacked a specific subtype designation even when categorized as CNS embryonal tumors. These discrepancies suggest a potential histologic overlap with other high-grade CNS tumors, leading to misclassification when relying solely on histopathology. While prior studies have proposed immunohistochemical markers for the diagnosis of CNS NB-FOXR2^12^ (including OLIG2, SOX10, TTF-1, and ANKRD55), immunoprofiles can be variable, and the markers are non-specific. Our findings highlight the critical role of molecular diagnostics, particularly DNA methylation profiling in accurately identifying CNS NB-FOXR2.

Accurate and prompt upfront diagnosis is key, as it dictates appropriate treatment planning. Limited data suggest that CNS NB-FOXR2 tumors generally respond well to therapy and may be associated with favorable outcomes, in contrast to high-grade gliomas.^14^^,^  ^23^^,^^24^ Notably, 4 of 5 patients in the cohort were initially diagnosed prior to the discovery of CNS NB-FOXR2. These patients had highly disparate and variable treatment courses, multiple lines of therapy, and multiple recurrences. With the caveat of a small sample size, one patient had accurate upfront diagnosis due to the availability of molecular testing. This patient had the least number of treatment regimens without tumor recurrence, highlighting the critical importance of utilizing molecular testing early in the diagnostic process. This is particularly important for patients seen at local community hospitals, where access to molecular pathology and specialized pediatric care, including pediatric neuropathologists, neurosurgeons, radiation oncologists, and pediatric neuro-oncologists, may be limited. Given the rarity of CNS NB-FOXR2, we recommend a diagnostic workflow that integrates histopathology, immunohistochemistry, and molecular profiling, including DNA methylation profiling, particularly for pediatric supratentorial tumors with ambiguous histology.

By copy number variant analysis, all CHLA cases in our cohort demonstrated 1q gain. Also consistent with Sturm et al, 4 cases showed loss of 16q and 2 demonstrated gain of chromosome 8.^7^ Additionally, all 3 female patients showed X chromosome loss in the tumor. While individual CNVs may not be specific, the overall pattern, 1q gain, 16q loss, 11q loss, chromosome 8 gain, and loss of X in females, may be characteristic of CNS NB-FOXR2 and should prompt consideration of this entity in the differential diagnosis. The loss of X chromosome in female patients is notable given that *FOXR2* is located on the X chromosome. Although the mechanism is unclear, this may have implications for *FOXR2* regulation or expression. A slight female predominance in CNS NB-FOXR2 has been previously documented,^7^^,^^12^ suggesting possible sex-specific biological factors. The loss of the X chromosome in the tumor could potentially unmask regulatory disruption on the remaining X, but further studies are needed to explore this.

The molecular mechanisms underlying CNS NB-FOXR2 alterations involve a high level of structural complexity leading to disruption of the upstream regulatory region of *FOXR2*. This region can be altered in multiple ways and at several breakpoints, including the previously annotated Exon -3.^3^ Our findings, along with previously published studies, demonstrate that these structural alterations are heterogeneous and non-recurrent, with each case showing a distinct genomic event, including insertion, inversion, tandem duplication, deletion, and complex rearrangement. In our cohort specifically, the breakpoints were located 25 kb, 65.7 kb, and 17.98 kb upstream of *FOXR2*. Despite this variability, all breakpoints appear to be consistently located within approximately 150 kb upstream of *FOXR2*, a regulatory zone that is vulnerable to genomic disruptions leading to *FOXR2* activation. It is unclear whether the exact location of the breakpoint influences the level or pattern of *FOXR2* expression in this tumor type. Importantly, these structural alterations do not affect the coding region of *FOXR2* and may not result in RNA fusion transcripts or chimeric proteins. Instead, *FOXR2* overexpression may occur through mechanisms such as promoter hijacking, which require direct assessment of gene expression. Given that *FOXR2* may not be included in existing oncology panels and that routine clinical sequencing approaches typically focus on exonic regions, these abnormalities are unlikely to be detected through standard clinical testing. Accurate identification of such abnormalities on the DNA level requires comprehensive genomic profiling techniques, such as OGM, SV calling from whole genome sequencing, or long-read sequencing. These methods are not commonly available in routine clinical laboratories and are generally considered specialized rather than mainstream diagnostic approaches, particularly for solid tumors.

Finally, *FOXR2* expression is not exclusive to CNS NB-FOXR2. Multiple other CNS tumors harbor *FOXR2* activation, as observed in our study and published reports.^14^ This highlights the need for caution when interpreting *FOXR2* expression alone, as it may not be specific or diagnostic. Future work is needed to delineate the spectrum of *FOXR2* alterations across different tumor types and clarify their biologic significance.

## Supplementary Material

wuag014_Supplementary_Data

## Data Availability

The data used in this study are derived from clinical cases and are not publicly available due to patient privacy and institutional restrictions. However, de-identified research data, including optical genome mapping results, may be made available upon reasonable request to the corresponding authors.
