# Supplementary figures and images for: FOXR2-activated CNS neuroblastoma: Characterized by variable structural disruption of the FOXR2 regulatory region, recurrent copy number alterations, and elevated FOXR2 expression
Source: Neurooncol Pediatr. 2026 Mar 19;2(2):wuag014. doi: 10.1093/neuped/wuag014 (PMC13070476; doi:10.1093/neuped/wuag014)

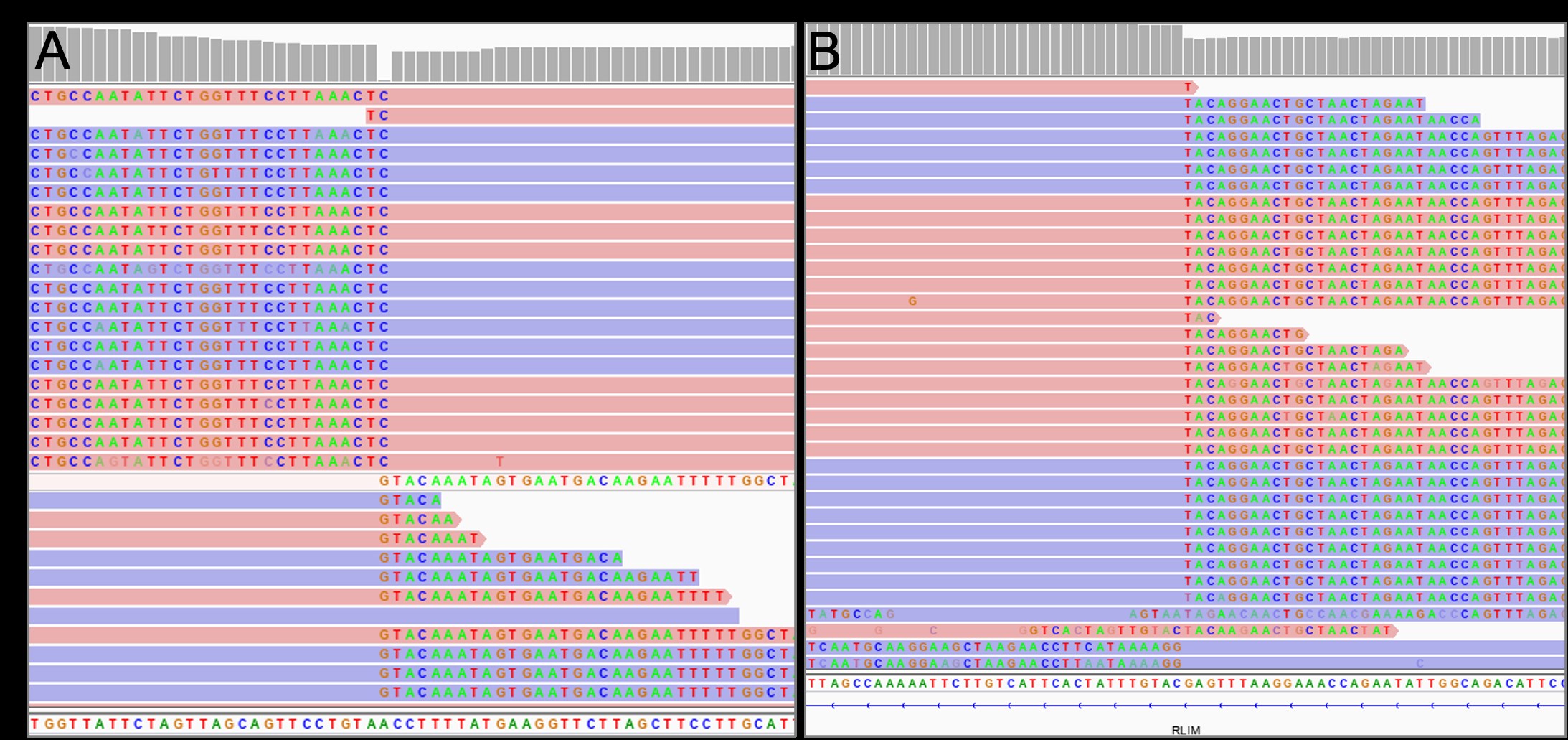

Supplement: wuag014_Supplementary_Data [file wuag014_supplementary_data.jpeg]
